# Giant Cell Tumor of the Cranial Vault: A Rare Frontotemporal Case With Complete Neurological Recovery and Narrative Review

**DOI:** 10.7759/cureus.105596

**Published:** 2026-03-21

**Authors:** Anthuan Hazkour, Héctor Aceituno, Juan Lopéz-Urdaneta, Juan J Valero Quintero, Luisana Maldonado-De Santiago, Karen Cobeña-Macias, José Villamediana-Rodríguez, Francisco Rico-Fernández

**Affiliations:** 1 Neurosurgery, Hospital San Juan de Dios de Curicó, Curicó, CHL; 2 Neurosurgery, Instituto Clínico La Florida, Caracas, VEN; 3 General Medicine, Hospital San Juan de Dios de Curicó, Curicó, CHL; 4 Pathological Anatomy, Hospital San Juan de Dios de Curicó, Curicó, CHL

**Keywords:** calvarial tumor, cranial vault, denosumab, fronto-temporal tumor, giant cell tumor of bone, neurosurgery

## Abstract

Giant cell tumor of bone (GCTB) represents less than 1% of primary bone tumors, with cranial localization being exceptionally rare, accounting for 0.5-1% of all GCTB cases. Within cranial GCTB, the vast majority arise from endochondral skull-base bones (sphenoid, temporal, and clivus), while true calvarial involvement of membranous bones represents only 4% of cases. We present an 18-year-old male with a frontotemporal calvarial GCTB who underwent successful surgical resection with complete neurological recovery despite postoperative hemiparesis. The tumor demonstrated classic histopathological and immunohistochemical features, including CD68 positivity, approximately 10% Ki-67 proliferative index, and osteoclast-like multinucleated giant cells. Following gross total resection with wide margins to healthy dura and cranioplasty reconstruction, the patient experienced complete resolution of motor and speech deficits within six weeks through intensive rehabilitation. Postoperative magnetic resonance imaging confirmed complete tumor removal with no evidence of residual disease. This case is discussed within the context of a comprehensive narrative review highlighting the distinct biological behavior of calvarial GCTB compared to skull-base lesions, the critical importance of achieving gross total resection, and the emerging role of molecular diagnosis (H3F3A G34W mutation) and adjuvant therapies including denosumab. Calvarial GCTB offers superior surgical accessibility and prognosis compared to skull-base counterparts, with appropriately aggressive resection typically achieving cure without need for radiotherapy, thereby avoiding the well-documented risk of radiation-induced malignant transformation.

## Introduction

Giant cell tumor of bone (GCTB) is a benign but locally aggressive primary bone neoplasm that preferentially affects the epiphyseal-metaphyseal region of long bones, most commonly around the knee, in young adults during the third and fourth decades of life [1-5]. Despite its benign histology, GCTB demonstrates significant local destructive potential with recurrence rates ranging from 10% to 50% depending on treatment modality, and possesses the remarkable capacity for pulmonary metastasis (2-5% of cases) and malignant transformation (particularly following radiotherapy) [3-8].

Cranial involvement by GCTB is extraordinarily rare, constituting less than 1% of all cases, with most large series reporting skull involvement in only 0.5-1% of patients [2,4,5,8-10]. Their infrequent occurrence, combined with radiologic overlap with other osteolytic skull lesions, may pose significant diagnostic challenges. Within this already uncommon cranial subset, a marked anatomical predilection exists for endochondral skull-base bones-specifically the sphenoid body (47%), petrous-mastoid portion of the temporal bone (28%), clivus (12%), and occipital bone (9%), while calvarial vault involvement (frontal and parietal bones) accounts for a mere 4% of cranial GCTB [2,4,5,8,11,12].

This distinctive anatomical distribution reflects fundamental embryological differences: skull-base bones develop through endochondral ossification (similar to long bones where GCTB typically arises), whereas the calvarium develops through intramembranous ossification. Consequently, calvarial GCTB represents not only a statistical rarity but also a biologically intriguing departure from the typical behavior of this tumor [5,11,13,14].

Recent molecular advances have revolutionized the diagnosis and management of GCTB. The identification of the H3F3A p.G34W mutation as a pathognomonic marker of GCTB, demonstrable by immunohistochemistry for mutant histone H3.3, has enabled definitive distinction from morphological mimics, including central giant cell granuloma, aneurysmal bone cyst, chordoma, and chondrosarcoma, a particularly crucial differential in the skull-base region [2]. Furthermore, the demonstration of robust RANKL overexpression in GCTB, including cranial cases, has provided the biological rationale for targeted therapy with denosumab (a fully human monoclonal antibody against RANKL), which has emerged as a major therapeutic adjunct for unresectable, recurrent, or surgically challenging skull-base tumors [2,6,15-20].

Several knowledge gaps persist regarding GCTB of the cranial bones. These include a lack of understanding of the risk factors and pathogenic mechanisms underlying tumor development in bones formed through intramembranous ossification, which challenges the classical endochondral ossification paradigm [3-5,11]; the absence of systematic molecular characterization (H3F3A G34W mutation profile and epigenetic alterations) specifically in cranial locations, as nearly all available evidence derives from long bones [2]; the unpredictability of biological behavior in the absence of validated prognostic factors to anticipate dural or parenchymal invasion [1,3-5]; the nonspecificity of CT and MRI imaging findings, which precludes a reliable differential diagnosis without biopsy [4,5,9,14]; the uncertainty surrounding the role of denosumab in this location, including optimal treatment duration, risk of malignant transformation, and the paradoxical effect of tumor cell entrapment within newly formed bone [6,16,19,20]; the lack of site-specific surgical guidelines and follow-up protocols for cranial GCTB; and, fundamentally, an extremely limited evidence base that relies almost exclusively on isolated case reports, without prospective series or multicenter registries to support robust evidence-based recommendations [1,3,6,8].

We report a case of frontotemporal calvarial GCTB in an 18-year-old male who achieved complete neurological recovery following gross total resection despite immediate postoperative deficits. This case exemplifies the favorable prognosis associated with calvarial lesions compared to skull base GCTB. Furthermore, we present a comprehensive narrative review of the literature addressing epidemiology, clinical presentation, imaging characteristics, pathological features, including molecular diagnostics, surgical management strategies, the role of adjuvant therapies, and outcomes stratified by anatomical location. The aim of this study is to synthesize the current evidence on cranial GCTB, identify persisting knowledge gaps, and provide a clinically oriented framework to guide diagnostic and therapeutic decision-making in this rare entity.

## Case presentation

Clinical history and presentation

An 18-year-old Chilean male with no significant medical history presented with a two-month history of progressive left frontotemporal scalp swelling, first noted on September 15, 2025. The mass was associated with intermittent headache and nausea, both of which resolved spontaneously prior to medical evaluation. The patient denied visual disturbances, seizures, focal weakness, sensory changes, or constitutional symptoms. Physical examination demonstrated a firm, non-tender, non-pulsatile left frontotemporal mass with intact overlying skin. Neurological examination showed a Glasgow Coma Scale score of 15, with no focal neurological deficits, normal cranial nerve function, and symmetric motor strength and reflexes in all extremities. No clinically significant abnormalities were observed in vital signs or laboratory values at admission (Table 1).

**Table 1:** Vital signs and laboratory findings at admission. *Values outside the normal reference range. Laboratory parameters were included to rule out metabolic bone disorders, hyperparathyroidism, and other systemic conditions that may mimic or coexist with giant cell tumor of bone, as well as to establish a baseline prior to surgical intervention. Abbreviations: bpm, beats per minute; eGFR, estimated glomerular filtration rate; HPF, high-power field; MCHC, mean corpuscular hemoglobin concentration; MDRD, Modification of Diet in Renal Disease

Parameter	Value	Reference Range
Vital signs		
Blood pressure (mmHg)	120/60	< 120/80
Heart rate (bpm)	75	60-100
Respiratory rate (breaths/min)	18	12-20
Oxygen saturation, SpO₂ (%)	99	95-100
Complete blood count		
White blood cells (×10³/µL)	10.7	4.5-11.0
Red blood cells (×10⁶/µL)	4.4	4.0-5.2
Hemoglobin (g/dL)	13.3	12.0-16.0
Hematocrit (%)	39.2	36.0-46.0
Mean corpuscular volume (fL)	90.1	80.0-100.0
Mean corpuscular hemoglobin (pg)	30.6	26.0-34.0
MCHC (g/dL)	33.9	31.0-37.0
Platelet count (×10³/µL)	281.0	140.0-400.0
White blood cell differential (%)		
Neutrophils (segmented)	70.1	50.0-70.0
Lymphocytes	18.9	25.0-40.0
Monocytes	7.1	4.0-12.0
Eosinophils	3.0	2.0-4.0
Basophils	0.9	0.0-1.0
Band neutrophils	0.0	0.0-5.0
Serum biochemistry		
Glucose (mg/dL)	91	70-100
Blood urea nitrogen (mg/dL)	19	6-20
Urea (mg/dL)	41	10-50
Creatinine (mg/dL)	0.7	0.7-1.2
eGFR by MDRD (mL/min/1.73 m²)	144.5	≥ 60.0
Lactate (mg/dL)	10.8	4.5-19.8
C-reactive protein (mg/dL)*	1.17	0.00-0.50
Electrolytes		
Sodium (mEq/L)	142.0	136.0-145.0
Potassium (mEq/L)	3.8	3.5-5.1
Chloride (mEq/L)*	111.0	96.0-106.0
Calcium (mg/dL)	10.0	8.1-10.4
Phosphorus (mg/dL)*	4.7	2.7-4.5
Urinalysis		
Epithelial cells	Scant/HPF	Scant/HPF
Leukocytes	0-2/HPF	0-5/HPF
Erythrocytes	0-2/HPF	0-3/HPF
Crystals	Not observed	Not observed
Casts	Not observed	Not observed
Bacteria	Scant/HPF	None-scant/HPF
Yeast	Not observed	Not observed

Neuroimaging findings

**Figure 1:**
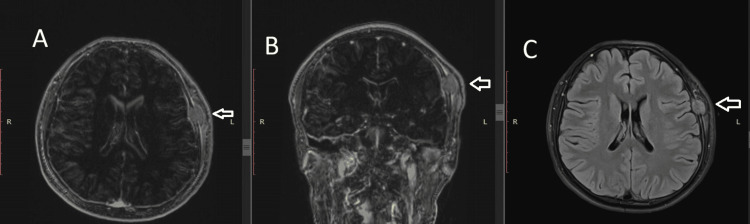
Preoperative brain MRI. (A) Axial T1-weighted post-contrast image demonstrating a moderately hypointense, partially circumscribed osteolytic lesion arising from the frontotemporal bone with intracranial extension, measuring 16 × 14 × 22 mm (AP × transverse × craniocaudal) (arrow). (B) Coronal T1-weighted post-contrast image showing the lesion's relationship to the frontotemporal bone, with osseous destruction and epidural extension (arrow). (C) Axial T2-FLAIR image revealing an isointense lesion without perilesional edema, consistent with an extra-axial compressive process without parenchymal invasion (arrow). FLAIR, fluid-attenuated inversion recovery

Magnetic resonance imaging with gadolinium contrast performed on November 5, 2025, revealed an aggressive extraaxial expansile mass centered in the left frontotemporal region measuring 16 mm anteroposterior × 14 mm transverse × 22 mm craniocaudal, demonstrating the following characteristics: partial destruction of the diploic space of both the outer and inner tables of the calvarium; infiltrative extension into the subjacent subaponeurotic fat plane; invasion of the adjacent left temporalis muscle; and heterogeneous contrast enhancement. Notably, there was no evidence of underlying brain parenchymal invasion or significant mass effect on the frontal lobe. The radiological differential diagnosis included atypical intraosseous meningioma, Langerhans cell histiocytosis, and hemangiopericytoma, with GCTB not initially suspected, given the unusual calvarial location (Figure 1).

Surgical management

Following neurosurgical consultation at Hospital San Juan de Dios de Curicó, surgical resection was recommended. The patient underwent an operation on December 16, 2025, under general anesthesia. A curvilinear left pterional incision was performed with systematic dissection through tissue planes. Intraoperative findings confirmed an infiltrative lytic lesion involving the left frontal bone with extension into the temporalis muscle. Intraoperative neurophysiological mapping was not deemed necessary given the extra-axial nature of the tumor and the absence of involvement of eloquent cortical areas.

Motorized craniectomy of the involved left frontal bone was performed with margins extending to macroscopically normal bone. Complete durotomy was undertaken with surgical margins of 0.5 cm to grossly uninvolved dura. Meticulous inspection confirmed no invasion of the underlying brain parenchyma. Tissue specimens were obtained from three distinct anatomical compartments for histopathological analysis: (1) involved bone, (2) infiltrated dura mater, and (3) affected temporalis muscle.

Following tumor resection, meticulous hemostasis was achieved using Surgicel® (Ethicon®, Somerville, NJ) (oxidized regenerated cellulose) and Surgiflo® (Ethicon®, Somerville, NJ) (hemostatic matrix). Duraplasty was performed using a collagen-based dural graft matrix (DuraGen®, Integra LifeSciences Corporation, Princeton, NJ). Cranial reconstruction was accomplished using a synthetic hydroxyapatite bone cement (Mimix®, Zimmer Biomet, Jacksonville, FL), secured with 6 mm titanium microscrews. Copious irrigation was performed, hemostasis reverified, and the wound closed in layers. The procedure was completed without intraoperative complications with an estimated blood loss of 400 mL.

Postoperative course and rehabilitation

**Figure 2:**
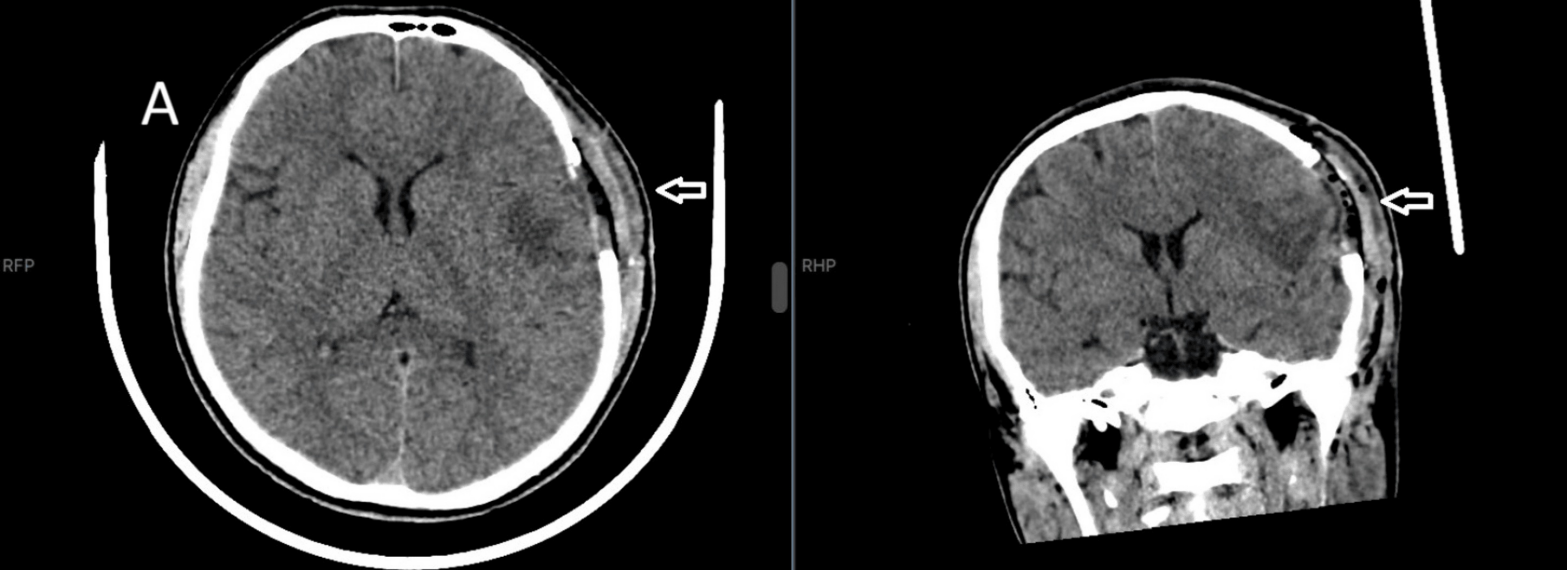
Postoperative non-contrast CT scan. (A) Axial view demonstrating post-surgical changes following left frontotemporal craniectomy for intra-axial lesion resection. The arrow indicates the left frontotemporal surgical cavity, measuring approximately 3.7 × 3.3 cm, with residual hemorrhagic content and perilesional white matter edema. (B) Coronal view; the arrow points to the left frontotemporal surgical cavity, showing surrounding vasogenic edema and a thin layer of subarachnoid hemorrhage. An extradural hydroaeric collection is noted adjacent to the craniectomy defect, likely containing hemostatic material.

In the immediate postoperative period, the patient developed right-sided hemiparesis (Medical Research Council grade 3/5) accompanied by mild dysarthria. Computed tomography performed on postoperative day 1 (December 17, 2025) demonstrated a left frontotemporal craniectomy with regular margins measuring 3.7 × 3.3 cm in diameter on bone window imaging, along with expected postoperative changes including mild perilesional edema, without evidence of hemorrhage or infarction, suggesting that the deficits were likely attributable to transient cerebral edema and surgical manipulation of the motor cortex during dural resection near the precentral gyrus (Figure 2). 

**Figure 3:**
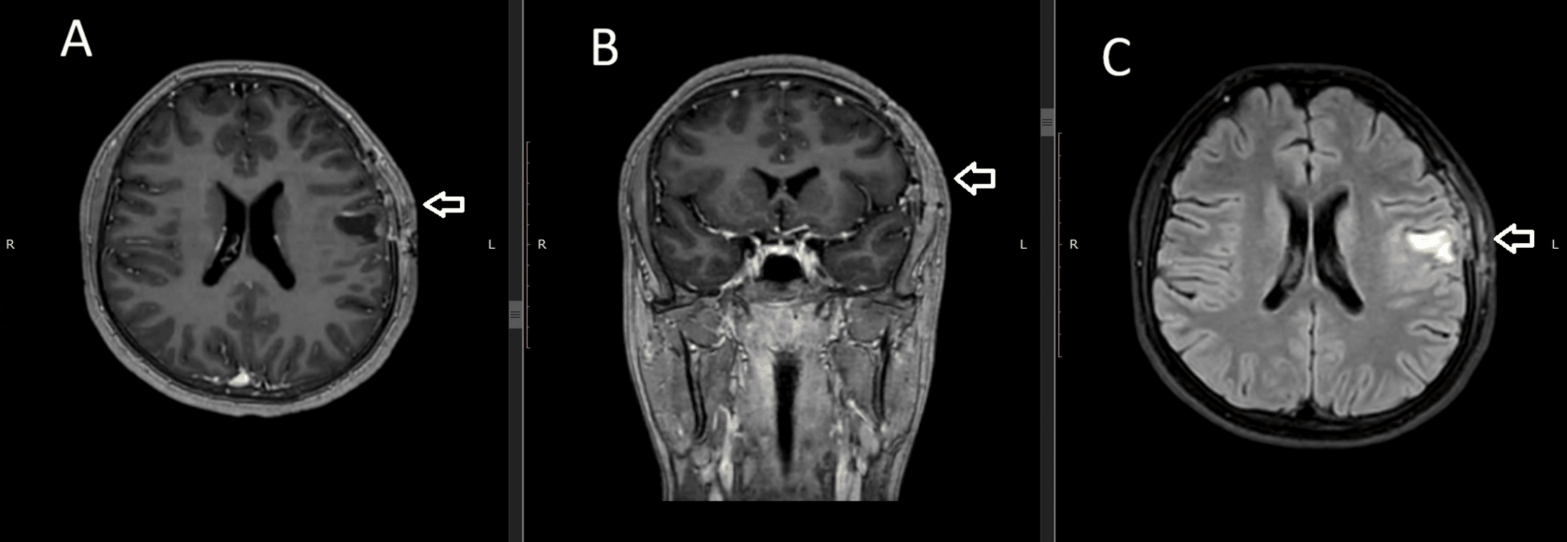
Postoperative brain MRI at two-month follow-up. (A) Axial T1-weighted image and (B) coronal T1-weighted image demonstrating postoperative changes in the right frontotemporal region with no evidence of residual or recurrent tumor (arrows). The surgical craniectomy defect is visualized. (C) Axial T2-FLAIR image showing expected postoperative gliosis and encephalomalacia at the surgical site (arrow). FLAIR, fluid-attenuated inversion recovery

Postoperative magnetic resonance imaging with gadolinium contrast performed during early follow-up (January 30, 2026) demonstrated postoperative changes in the left frontoparietal region with a surgical cavity of slightly smaller dimensions compared to the preoperative tumor volume. Linear enhancement along the adjacent margins and cortex was observed, consistent with expected postoperative enhancement patterns. Diffusion-weighted imaging (DWI) showed no evidence of restricted diffusion, excluding acute ischemic injury. Critically, no nodular enhancement or thickening suggestive of residual tumor was identified, confirming gross total resection (Figure 3).

The patient's hospital course was otherwise uncomplicated, and he was discharged on postoperative day 4 with referrals for intensive physical therapy and speech therapy. A comprehensive rehabilitation program was initiated immediately upon discharge, incorporating daily physical therapy focusing on strength, coordination, and gait training, as well as speech therapy addressing articulation and fluency.

The patient demonstrated progressive improvement in motor function and speech with remarkable neuroplasticity. By six weeks postoperatively, complete resolution of both hemiparesis and dysarthria was achieved, with restoration of normal strength (MRC grade 5/5) in the right upper and lower extremities and normalized speech fluency. At three-month follow-up, the patient remained neurologically intact with a well-healed surgical incision and no clinical or radiological evidence of tumor recurrence. A surveillance protocol was established, consisting of magnetic resonance imaging every three months during the first year and every six months thereafter, with a minimum planned follow-up of three years, to monitor for potential recurrence. Long-term imaging surveillance is necessary given the reported recurrence rates of GCTB.

Histopathological and immunohistochemical analysis

**Figure 4:**
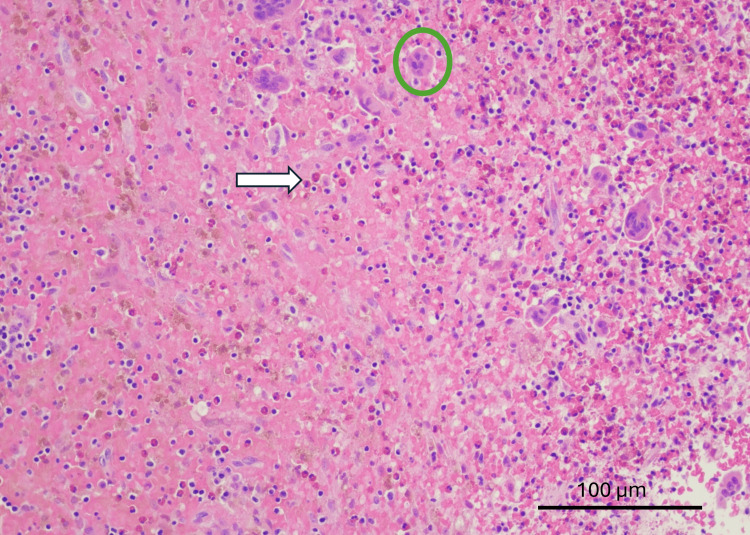
Numerous multinucleated giant cells (circle) associated with an inflammatory infiltrate (arrow) composed of histiocytes, lymphocytes, and eosinophils. No atypia, necrosis, or mitotic figures are identified. Original magnification ×20; scale bar = 100 μm.

**Figure 5:**
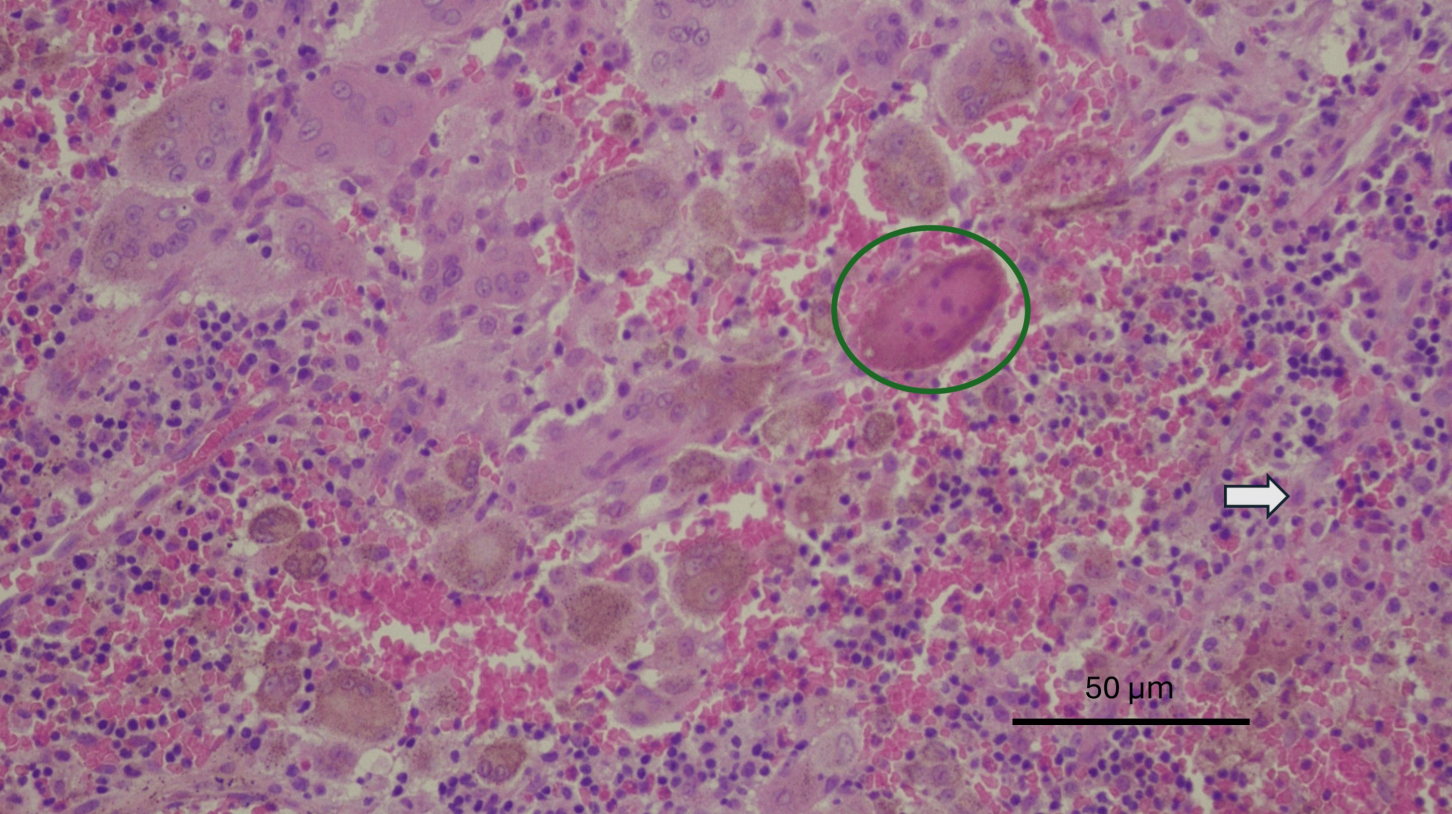
Higher magnification demonstrates the intimate relationship between osteoclast-type multinucleated giant cells (circle) and mononuclear stromal cells (arrow), which exhibit round to oval morphology with scant cytoplasm and uniform nuclei. No significant cytologic atypia, necrosis, or atypical mitotic figures are identified in the evaluated sections. Original magnification ×40; scale bar = 50 μm.

Histopathological examination (reported January 2026) revealed the classic diagnostic features of GCTB: bone tissue with extensive proliferation of mononuclear stromal cells with ovoid nuclei, uniformly intermixed with osteoclast-like multinucleated giant cells whose nuclei were morphologically similar to those of the surrounding stromal cells. No osteoid production was identified, further supporting the diagnosis and distinguishing it from other giant cell-rich bone lesions. The stromal cells displayed mild-to-moderate nuclear atypia without significant pleomorphism or mitotic activity. There was no evidence of necrosis, atypical mitotic figures, or sarcomatous transformation (Figures 4-5).

**Figure 6:**
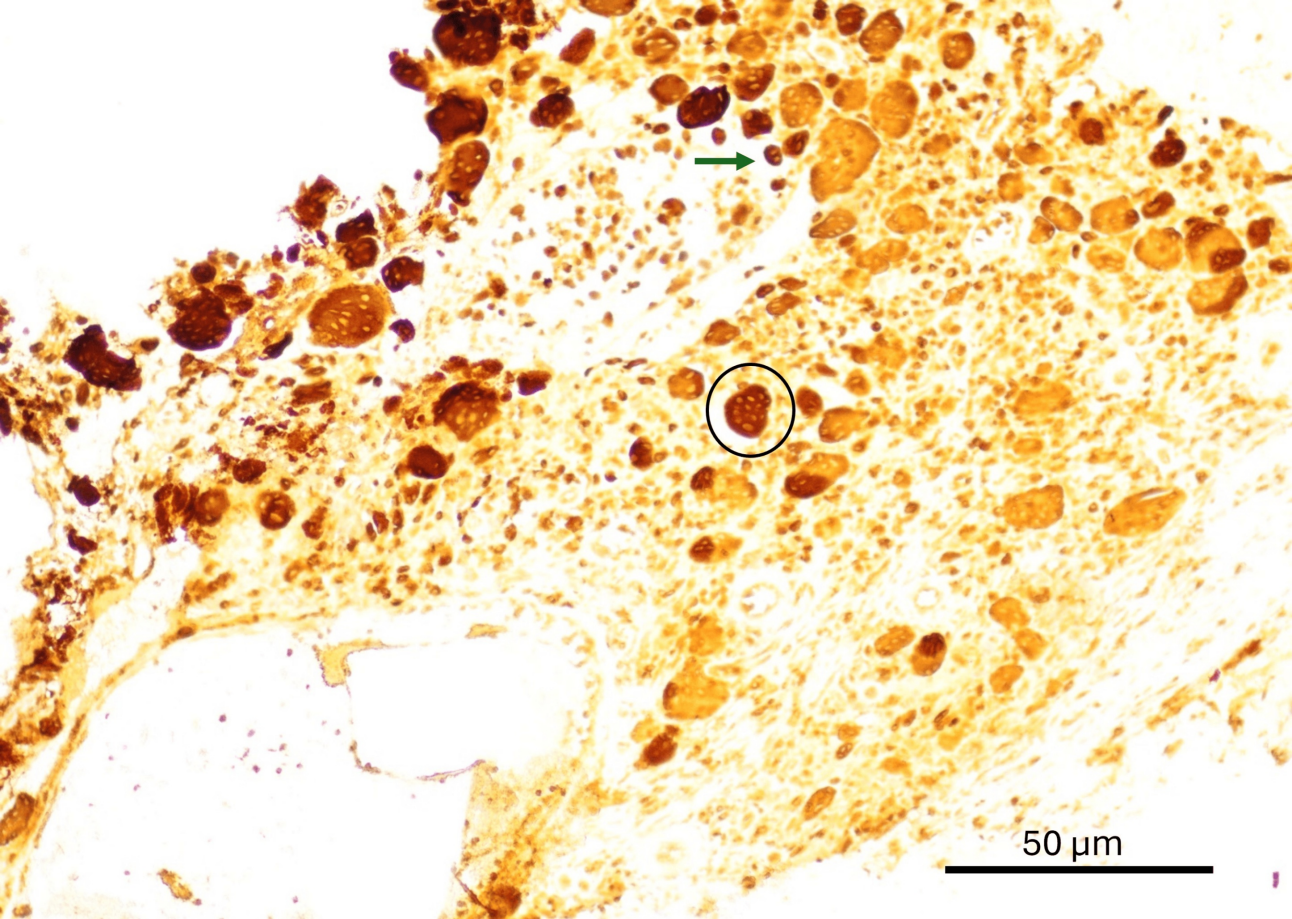
Immunohistochemical staining for CD68 demonstrates strong positivity in multinucleated giant cells (circle) and variable cytoplasmic staining in mononuclear cells (arrow), supporting the non-epithelial nature of the proliferation. Original magnification ×40; scale bar = 50 μm.

Immunohistochemical studies demonstrated the following profile: CD68 strongly positive in the cells under study, confirming their histiocytic/osteoclastic lineage; S100 positive in a subset of cells; GFAP negative, excluding glial origin; Ki-67 proliferative index of approximately 10%, indicating moderate proliferative activity within the benign range; and CD45 positive in a subset of cells (Figure 6).

This immunophenotype, in conjunction with the characteristic histomorphology, established the diagnosis of giant cell tumor of bone. 

A limitation of this case is the absence of H3F3A G34W mutation analysis, which was unavailable at our institution. However, the diagnosis was established based on characteristic histomorphological features and a supportive immunohistochemical profile (CD68+, vimentin+, p63+, Ki-67 <10%), which are considered sufficient for a definitive diagnosis of GCTB, particularly in resource-limited settings [2].

## Discussion

Epidemiology and anatomical distribution of cranial GCTB

The present case exemplifies the extreme rarity of calvarial GCTB. Across comprehensive GCTB datasets, skull involvement occurs in less than 1-2% of all cases, with skull cases representing approximately 0.5-1% of the total GCTB burden [2,4,5,8-10]. Weng and colleagues pooled 133 intracranial giant cell tumors (institutional series plus literature review), confirming their exceptional rarity relative to appendicular disease [1].

Within cranial GCTB, there exists a marked predilection for skull-base endochondral bones. The present frontotemporal case, therefore, represents an unusual variant occurring in the junction between membranous (frontal) and endochondral (temporal squamous) bone. The most quantitative skull-base distributions derive from Scotto di Carlo's systematic review of 104 skull-base cases (1969-2017) and Freeman's lateral skull-base meta-analysis of 67 patients [2,3]. Scotto di Carlo reported the following anatomical distribution: sphenoid 47%, temporal 28%, clivus 12%, occipital 9%, and frontal only 4% [2]. These data consistently demonstrate a strong bias toward endochondral skull-base bones, with calvarial frontal and parietal lesions being distinctly rare [4,5,8,11,12,21]. 

Cranial GCTB generally mirrors the age distribution of conventional GCTB, demonstrating a young adult peak in the second to fourth decades [2,4,5,8-10]. Clival and sphenoid series report mean ages in the mid-twenties, with well-documented pediatric cases ranging from nine to 16 years [6,7,10,16,17,19,20]. Most series describe a slight female predominance overall, though small cranial cohorts often appear balanced (for example, among 27 clival cases, 15 were male, and 12 female [6]; in temporal series, distributions have been reported as 4M:3F [22] and 2M:4F [23]). Our 18-year-old male patient falls within the expected age range for both conventional and cranial GCTB.

Table 2 provides a comprehensive overview of previously reported cranial GCTB cases, detailing their anatomical location, clinical presentation, treatment approach, follow-up duration, and prognosis.

**Table 2:** Clinical manifestations, treatment, and follow-up of cranial giant cell tumor of bone by anatomical location Abbreviations: CN, cranial nerve; CPA, cerebellopontine angle; EAC, external auditory canal; GCTB, giant cell tumor of bone; IAC, internal auditory canal; ICA, internal carotid artery; MRI, magnetic resonance imaging; PMMA, polymethylmethacrylate; TMJ, temporomandibular joint

Anatomical Location	Clinical Manifestations	Treatment	Follow-Up and Prognosis
Calvarium (frontal, parietal) ~4% of cranial GCTB [2,4,5,8,11,12]	Localized scalp swelling and tenderness [11,12]; headache; focal neurological deficits only if dural/brain invasion occurs [11,12]. Generally detected earlier due to visible external mass effect [11,12]. Present case: frontotemporal lesion with dural involvement but no preoperative neurological deficit.	Gross total resection is the treatment of choice given a superficial, surgically accessible location [11,12]. Dural resection and cranioplasty may be required. Intralesional curettage with adjuvants (high-speed burr, phenol, PMMA, hydrogen peroxide) as an alternative [3,5]. Denosumab is considered for incompletely resected or recurrent cases [6,15-20].	Favorable prognosis compared to skull-base GCTB [11,12]. MRI every three months during the first year, then every six months, with a minimum follow-up of three years [3,5]. Recurrence rates 10-50% depending on resection extent [3-8]. Monitor for late local recurrence and rare pulmonary metastasis (2-5%) [3-8].
Temporal Bone (petrous-mastoid) ~28% of cranial GCTB [2,4,5,8]	Hearing loss (nearly universal) [8,9,22,24,25]; otalgia; aural fullness; tinnitus; vertigo. Facial nerve palsy in perigeniculate/IAC-adjacent lesions [24,25]. Extension to mastoid, middle ear, EAC, middle fossa floor, and CPA TMJ/glenoid fossa involvement: preauricular swelling, TMJ pain, trismus, jaw locking [23-30].	Maximal safe resection; extent limited by involvement of critical neurovascular structures (facial nerve, ICA, sigmoid sinus) [8,24,25,28,29]. Staged procedures may be necessary. Denosumab as neoadjuvant to reduce tumor volume or as adjuvant for residual/recurrent disease [6,15-20]. Radiotherapy reserved for unresectable cases due to malignant transformation risk [3-8].	Serial MRI surveillance; recurrence risk is higher with subtotal resection [3-8]. Long-term audiological monitoring. Monitor for facial nerve function recovery. If denosumab is used: surveillance for rebound tumor growth after discontinuation [6,16,19,20].
Sphenoid bone (body, greater wing) ~47% of cranial GCTB [2,4,5,8]	Subacute headache; diplopia from CN III, IV, or VI involvement; visual loss or field defects from optic canal/chiasmal compression; facial hypoesthesia (V2) from trigeminal compression; nasal obstruction or epistaxis via sphenoid sinus extension [18,31]. Extension to sella turcica, cavernous sinus, orbit, clivus, and nasopharynx [7,10,17,18,31,32,33,34].	Complete resection is often unachievable due to cavernous sinus/ICA involvement [7,10,17,18,31-33]. Endoscopic endonasal approaches for selected cases. Denosumab as primary therapy or neoadjuvant for unresectable tumors [6,15-20]. Stereotactic radiosurgery for residual disease in selected cases [3-8].	Serial MRI with attention to cavernous sinus and optic apparatus [7,10,17,18]. Endocrinological evaluation if sellar extension is present. Higher recurrence risk due to frequent subtotal resection [3-8]. Long-term ophthalmological follow-up.
Clivus ~12% of cranial GCTB [2,4,5,8]	Headache (most frequent initial symptom); diplopia, particularly abducens palsy (CN VI); involvement of CN V, VII, and IX-XII [6,7,10,17,19,32]. Less commonly: optic neuropathy; pituitary dysfunction. Large lesions: brainstem compression with ataxia or long tract signs [6,7,10,17,19,32].	Surgical resection via transcranial or endoscopic endonasal approaches; complete resection is rarely feasible [6,7,10,17,19]. Denosumab as first-line for unresectable/recurrent tumors or neoadjuvant strategy [6,15-20]. Proton beam therapy as an alternative for residual disease [3-8].	Serial MRI with focus on brainstem and cranial nerves [6,7,10,17,19]. Cranial nerve function monitoring. The highest complexity for surveillance due to deep location and proximity to vital structures. Minimum five-year follow-up recommended given high recurrence potential [3-8].
Occipital bone (including condyle) ~9% of cranial GCTB [2,4,5,8]	Occipital headache; neck pain; palpable occipital mass [34-36]. Occipital condyle syndrome: occipital pain with lower cranial nerve (IX-XII) palsies [34-36]. Posterior fossa symptoms from cerebellar/brainstem compression or venous sinus involvement [34,35,36].	Surgical resection; craniocervical junction stabilization may be required for condylar lesions [34-36]. Occipitocervical fusion if condylar destruction compromises stability. Denosumab for unresectable or recurrent disease [6,15-20].	Serial MRI surveillance including craniocervical junction [34-36]. Biomechanical assessment of craniocervical stability. Lower cranial nerve function monitoring. Long-term follow-up given recurrence risk [3-8].

Radiological characteristics and differential diagnosis

Cranial GCTB demonstrates consistent imaging characteristics across all sites. On computed tomography, lesions are typically lytic, expansile, often multiloculated, with cortical thinning and focal breach, usually lacking a sclerotic rim [2,4,8-10,29,30,36,37]. On magnetic resonance imaging, tumors are isointense to hypointense to gray matter on T1-weighted sequences and demonstrate variable T2 signal characteristics, often heterogeneously hyperintense, though some clival and temporal reports describe relative T2 hypointensity compared with chordoma [7-10,25,30]. Gadolinium enhancement is characteristically heterogeneous and solid [2,9,19,29,30,37].

The radiological differential diagnosis is challenging and site-dependent. Clival and sphenoid GCTB can mimic chordoma or chondrosarcoma; absence of chondroid matrix or classic chordoma signal characteristics is suggestive but not definitive [2,7,10,34]. Temporal bone lesions may resemble central giant cell granuloma, cholesteatoma, paraganglioma, or facial schwannoma [8,9,24,29,30]. In the present calvarial case, atypical intraosseous meningioma, Langerhans cell histiocytosis, and hemangiopericytoma were appropriately considered in the preoperative differential, illustrating the diagnostic challenge posed by this rare tumor in an uncommon location.

Histopathological features and molecular diagnosis

Across all cranial sites, the morphological features of GCTB match those of conventional appendicular lesions. Histologically, tumors demonstrate uniformly distributed osteoclast-like multinucleated giant cells within a background of mononuclear stromal cells exhibiting mild to moderate atypia [2,4,5,7-10,12,25,36]. Stromal mitotic activity may be brisk, but atypical mitoses and necrosis are absent in benign tumors. Sarcomatous transformation is characterized by overt cellular atypia and markedly elevated Ki-67 proliferative index [7,38,39]. Secondary aneurysmal bone cyst-like areas are common and can be extensive [2,19,30,34].

The major breakthrough in GCTB diagnosis has been the identification of the H3F3A p.G34W mutation as a molecular signature. Scotto di Carlo and colleagues demonstrated this mutation by sequencing and immunohistochemistry for mutant H3.3 in clival GCTB, with strong RANKL expression [2]. Subsequent clival series have confirmed G34W immunopositivity [6].

H3.3 G34W immunohistochemistry serves as a powerful diagnostic tool to distinguish GCTB from central giant cell granuloma and primary aneurysmal bone cyst (typically H3F3A-wild-type; ABC often demonstrates USP6 rearrangement), from chordoma (brachyury-positive, cytokeratin-positive), and from chondrosarcoma (demonstrates cartilaginous matrix; often harbors IDH1/2 or EXT alterations) [2].

RANKL overexpression in cranial GCTB provides the biological rationale for denosumab therapy [2,15,19,20]. In the present case, while molecular H3F3A testing was not performed, the characteristic histomorphology combined with the immunohistochemical profile strongly supported the diagnosis. The CD68 positivity confirmed the histiocytic/osteoclastic lineage of the giant cells, while the Ki-67 index of approximately 10% indicated moderate proliferative activity within the benign spectrum, consistent with typical GCTB behavior [7,38,39].

Surgical management and the primacy of the extent of resection

The fundamental principle governing cranial GCTB management is that the extent of resection represents the dominant determinant of local control across all cranial sites. Freeman's meta-analysis of 67 skull GCTB cases quantified this relationship: complete resection (with or without radiotherapy) resulted in only three of 34 recurrences (8.8%); subtotal resection followed by radiotherapy yielded three of 21 recurrences (14.3%); whereas subtotal resection alone produced seven of 10 recurrences (70%), representing approximately 14-fold higher recurrence risk compared to complete resection [3,21].

A clival dataset of 25 patients with adequate follow-up demonstrated that gross total resection was achieved in only six patients, none of whom experienced recurrence. Overall, nine of 25 patients (36%) recurred, all following subtotal resection, and notably, all patients undergoing subtotal resection without adjuvant therapy experienced recurrence [6].

Multiple temporal bone series with long-term follow-up (3-10+ years) report no recurrences after gross total resection [8,22,24,25,28,40]. However, Li's analysis of 46 temporal giant cell lesions still documented a 31.8% recurrence rate despite stage-tailored surgery, underscoring the aggressive biological potential of these tumors [30]. An occipital bone review of 22 analyzable cases reported overall recurrence rates approaching 30%, predominantly after incomplete resection [34].

Surgical approaches are necessarily site-specific. Temporal bone and lateral skull-base lesions are managed via infratemporal fossa approaches, petrosectomy variants, combined transmastoid and middle fossa approaches, and extended pterional or temporal craniotomies, permitting high gross total resection rates in many contemporary series [8,22-25,28-30]. Sphenoid and clival tumors are predominantly addressed through endoscopic endonasal or transsphenoidal approaches, often supplemented by transcranial routes for large lateral or posterior extension [8,22-25,28-30].

High vascularity and neurovascular encasement frequently limit resection to subtotal removal [6,7,10,15,17,18,20,31,32,37]. Occipital, foramen magnum, and occipital condyle lesions require suboccipital, far-lateral, or transcondylar approaches, with occipitocervical fusion potentially necessary when the condyle is substantially resected [34,35,41].

Calvarial lesions, such as the present case, are optimally managed by en bloc craniectomy with wide margins, which is usually feasible and potentially curative [11,12]. In our patient, motorized craniectomy of the involved frontal bone with margins extending to macroscopically normal bone, combined with durotomy with 0.5 cm margins to uninvolved dura, exemplifies the principle of achieving gross total resection with adequate margins-an approach that would be anatomically impossible in most skull-base locations. Postoperative imaging confirmed complete tumor removal with no evidence of residual disease, validating the surgical strategy. This fundamental surgical advantage explains the superior prognosis of calvarial GCTB compared to skull-base counterparts.

Role of radiotherapy: efficacy versus transformation risk

Radiotherapy has historically been employed for residual or unresectable cranial GCTB and clearly improves local control after subtotal resection relative to surgery alone. Freeman's pooled data demonstrated that subtotal resection plus radiotherapy yielded 14.3% recurrence versus 70% with subtotal resection alone [3,21]. Similar patterns emerge across skull-base and occipital case series [8,10,34,36]. Typical modern regimens employ 45-50 Gy in 1.8-2.0 Gy fractions with conformal planning; stereotactic radiotherapy and stereotactic radiosurgery have been utilized, though long-term data remain sparse [7,10,19,36].

However, multiple cranial reports document secondary sarcomas after radiotherapy, particularly in clival disease. Shibao and colleagues reported clival GCTB with rising MIB-1 (Ki-67) index and fatal progression after 50 Gy radiotherapy, interpreted as malignant transformation [7]. Sasagawa et al. documented secondary malignant clival GCTB following radiotherapy [38]. Yang et al.'s 2024 clival review identified two malignant transformations among 12 radiotherapy-treated patients [6]. Historical series of appendicular GCTB quote radiotherapy-associated malignant transformation rates ranging from 5% to 29%, and Isaacson et al.'s temporal bone review cited an overall malignant GCTB rate of approximately 1.8%, with shorter latency after radiotherapy (approximately nine years) compared to surgery alone (approximately 18 years) [8].

Current practice reserves radiotherapy for incompletely resected or unresectable lesions where further surgery is unsafe, patients ineligible for or refractory to denosumab, with decision-making being age-, site-, and risk-dependent. There exists a strong bias against radiotherapy in young patients when denosumab or re-resection is feasible [2,6,8,29,34,35].

In the present case of calvarial GCTB with gross total resection achieved, radiotherapy was appropriately not administered, eliminating the risk of radiation-induced malignant transformation while achieving cure through surgery alone treatment paradigm generally unavailable for skull-base lesions, where complete resection is rarely feasible.

Denosumab: emerging role in skull-base GCTB

The skull-base literature increasingly supports denosumab (anti-RANKL monoclonal antibody) as a major adjunct in management [6,15,18,19]. Clinical indications include neoadjuvant use to shrink and harden tumors while reducing vascularity before skull-base surgery. Inoue's juvenile clival GCTB case demonstrated that denosumab induced marked sclerosis and facilitated safer endonasal resection [20]. Multiple sphenoid and clival case series have integrated denosumab pre-operatively or peri-operatively [6,15,18,19].

Denosumab also serves as adjuvant or salvage therapy for residual or recurrent disease, particularly when radiotherapy is undesirable or has failed. Bardakhchyan et al. reported a dramatic response in a C1-C2 plus skull-base GCTB progressing after 50.4 Gy radiotherapy, with the patient experiencing significant clinical improvement after initiating denosumab [19]. Pionelli et al. documented long-term denosumab monotherapy (exceeding two years) controlling unresectable or recurrent clival GCTB in a 14-year-old without additional adjuvant treatment [16]. Combined surgery plus denosumab, with or without radiotherapy, in sphenoid and clival GCTB has been associated with reduced recurrence in small cohorts [15,17].

Observed effects include rapid symptomatic relief and radiologic regression or sclerosis in essentially all reported skull-base cases [6,15-20]. Control is often maintained while on therapy; however, at least one clival case relapsed after denosumab discontinuation, highlighting the need for cautious tapering and surveillance [6]. Pathologic changes following denosumab treatment complicate margin assessment: depletion of giant cells, increased fibrosis, and neobone formation can mask residual stromal cells [2,15,19,20].

In the cranial literature summarized in this review, no unequivocal denosumab-related malignant transformation has been documented, though follow-up remains short and sample sizes are small [6,15-20]. Broader GCTB data suggest possible transformation in a minority of denosumab-treated cases; therefore, long-term cranial-site surveillance remains essential [6,15-20].

In the present calvarial case, denosumab was not employed given successful achievement of gross total resection, which is the optimal scenario, obviating the need for systemic therapy.

Prognosis: recurrence, metastasis, and survival

Local recurrence patterns are consistent across cranial sites. Subtotal resection without adjuvant therapy demonstrates very high recurrence, often 40-70% within several years [3,6-8,10,32,35-37]. Subtotal resection plus radiotherapy substantially reduces recurrence to approximately 10-20%, at the cost of potential malignant transformation [3,8,10,29,34,36]. Gross total resection achieves recurrence rates generally under 10%, with many series reporting no recurrences among gross total resection patients with multi-year follow-up [3,8,21-25,28,29,35,40].

Site-specific nuances exist. Temporal bone and lateral skull-base lesions demonstrate the best prognosis, with multiple contemporary series showing durable control with gross total resection alone [8,22,24,25,40]. Under stage-adapted surgery, giant cell tumors still recurred in approximately 32% in Li et al.'s temporal cohort, signifying unforgiving biology when gross total resection is not fully achieved [30]. Clivus and sphenoid represent the highest-risk niche; gross total resection is rarely achievable, and clival datasets show overall recurrence of approximately 35-40%, entirely in subtotal resection patients [6,7,10,17,32].

Occipital and foramen magnum lesions show intermediate risk; reviews report recurrence up to approximately 30% overall, largely confined to incompletely resected tumors [34-36]. Calvarial lesions generally demonstrate low recurrence after complete excision [11,12], consistent with the favorable prognosis anticipated in our patient.

Pulmonary metastases remain uncommon but well-documented; skull and general GCTB series quote rates of approximately 2-5% [8-10,12,35]. Clival reviews identify rare lethal metastatic cases [17,32]. Metastatic deposits are often histologically benign (termed benign metastasizing GCTB) but may still be clinically serious. No consistent evidence suggests that cranial site intrinsically increases metastatic risk; most metastasizing cases have recurrent, long-standing disease with multiple local interventions [8,10,32].

Malignant transformation occurs as either primary malignant cranial GCTB (rare but exemplified by malignant GCTB of the parietal bone [39] and aggressive clival transformation without radiotherapy in Shibao et al.'s patient with MIB-1 index rising from 4.2% to 26.3% [7]) or secondary post-radiotherapy sarcoma, a recurring theme. Secondary malignant clival GCTB after radiotherapy was documented by Sasagawa et al. [38]. Clival series collectively report at least several malignant transformations, often following radiotherapy doses in the 45-50 Gy range [6,7,10,20,38]. Historical mixed-site GCTB series place post-radiotherapy malignant transformation risk in the range of several percent to tens of percent, depending on dose and era [8].

For histologically benign cranial GCTB, overall survival is generally excellent when local disease is controlled; deaths result from uncontrolled local progression (brainstem or cerebellar compression, raised intracranial pressure) [1,7,10,35] or rare metastatic spread, particularly from clival lesions [32]. Weng et al.'s 133-case intracranial giant cell tumor analysis identified extent of resection and adjuvant radiotherapy as key determinants of progression-free survival, emphasizing that achieving local control is synonymous with securing long-term survival in most cases [1]. In contrast, malignant cranial GCTB (primary or secondary) behaves as a high-grade skull-base or cranial sarcoma with poor outcomes despite aggressive multimodal treatment [7,38,39].

Neurological recovery and rehabilitation

The postoperative neurological deficits observed in our patient - right hemiparesis and dysarthria - likely resulted from transient manipulation or minimal injury to the motor cortex during resection of dura in proximity to the precentral gyrus, rather than from direct tumor invasion (which was not observed intraoperatively). The remarkable complete recovery within six weeks through intensive physical and speech therapy underscores the impressive neuroplastic capacity of the young adult brain. This favorable outcome further distinguishes calvarial from skull-base GCTB, where manipulation of critical neurovascular structures during attempted resection carries substantially higher risk of permanent cranial nerve deficits, vascular injury, or brainstem compromise. The accessibility and relative safety of calvarial resection, combined with the potential for complete tumor removal, position these rare lesions among the most favorable cranial GCTB variants from both an oncologic and functional perspective.

## Conclusions

This case of frontotemporal calvarial giant cell tumor of bone in an 18-year-old male underscores the exceptional rarity of GCTB arising from membranous cranial bones, which accounts for approximately 4% of all cranial cases. Gross total resection with wide surgical margins constitutes the primary prognostic factor and the cornerstone of curative treatment for calvarial GCTB, yielding superior oncologic outcomes compared to skull base lesions, where complete resection is frequently precluded by the proximity of critical neurovascular structures. The favorable surgical accessibility inherent to calvarial locations confers a distinct therapeutic advantage, rendering these lesions more amenable to definitive surgical management than their skull base counterparts.

The complete neurological recovery achieved within six weeks, despite immediate postoperative hemiparesis and dysarthria, illustrates the remarkable neuroplastic capacity of young adults and underscores the role of early intensive rehabilitation in optimizing functional outcomes. Notably, unlike skull base GCTB, where denosumab serves as a principal therapeutic modality for unresectable or residual disease, calvarial lesions amenable to gross total resection obviate the need for adjuvant pharmacological therapy or radiotherapy. This approach eliminates both the well-documented risk of radiation-induced malignant transformation and the uncertainties associated with prolonged denosumab administration, including paradoxical tumor cell entrapment within newly formed bone and the potential for sarcomatous transformation. This case contributes to the limited body of evidence on calvarial GCTB and reinforces the paradigm that appropriately aggressive surgical resection can achieve oncologic cure while preserving long-term functional outcomes.
